# Evaluating the Toxicity and Safety of Turmeric (*Curcuma longa* L.) and Its Components: A Narrative Review

**DOI:** 10.1155/jt/2715031

**Published:** 2026-07-21

**Authors:** Jalileh Jalali, Mahboobeh Ghasemzadeh Rahbardar

**Affiliations:** ^1^ Pharmacological Research Center of Medicinal Plants, Basic Sciences Research Institute, Mashhad University of Medical Sciences, Mashhad, Iran, mums.ac.ir; ^2^ Shahid Hasheminejad Hospital, Mashhad University of Medical Sciences, Mashhad, Iran, mums.ac.ir

**Keywords:** apoptosis, curcumin, DNA damage, mutagens, oxidative stress, pregnancy, teratogens

## Abstract

Turmeric (*Curcuma longa*), a widely used botanical in traditional medicine, has gained global attention for its therapeutic potential, particularly due to its components including curcumin. While its pharmacological benefits are extensively documented, concerns regarding its safety profile and potential toxicity have emerged, especially with high‐dose supplementation and long‐term use. This narrative review aims to critically evaluate the toxicological evidence and safety considerations surrounding turmeric and its main components. A comprehensive literature search was conducted across PubMed, Scopus, Web of Science, and Google Scholar, covering studies published up to October 2025. In vitro and in vivo studies were carried out to capture a broad spectrum of toxicological data. Toxicological profile of turmeric is influenced by dose, formulation, and metabolic context. Most turmeric‐based preparations exhibit low acute toxicity and minimal genotoxic or mutagenic effects under standard conditions. However, high concentrations or certain formulations, particularly crude (unfractionated) extracts, have been reported to induce oxidative stress and DNA damage in vitro, while some nanoparticle systems show mutagenic potential under metabolic activation conditions. In vivo studies generally report favorable safety outcomes, especially for standardized and bioavailable formulations, although some evidence of liver enzyme alterations and histopathological changes has been observed at supratherapeutic doses. Mechanistic insights implicate oxidative stress, mitochondrial dysfunction, and p53‐mediated pathways in the observed toxic effects. Turmeric and its components are generally safe when used at recommended doses and in well‐designed formulations. However, high‐dose or prolonged use may pose hepatic and genomic risks. Refining safety thresholds through harmonized methods and human‐relevant models will be the key to guiding their safe and effective clinical application.

**Highlights**
• Turmeric shows low acute toxicity across most tested formulations.
• High doses may trigger liver stress and DNA damage in some models.
• Genotoxicity depends on dose, formulation, and metabolic context.
• Lipid‐based and polysaccharide forms show better safety profiles.
• Human‐relevant models are needed to refine clinical safety thresholds.

## 1. Introduction

*Curcuma longa* Linn., commonly known as turmeric, is a perennial species with rhizomes and soft, leafy stems. It produces flowers and belongs to the Zingiberaceae family. This plant is native to regions across India and Southeast Asia [1], as well as Africa [2]. Its underground rhizomes, often referred to as roots, are widely used in cooking to add flavor and color. Beyond its role in the kitchen, turmeric has drawn attention from multiple fields such as medicine [1].

*C. longa* has long held a respected place in folk medicine, valued for its healing properties across generations. In Persian traditional medicine, turmeric, referred to as “Uruq al‐Ṣabbāghīn” in classical texts, is described by Avicenna in The Canon of Medicine (Al‐Qanun fi al‐Tibb) as having a warm and dry temperament. In the humoral framework of Persian medicine, substances are classified according to qualitative properties, heat, coldness, moisture, and dryness, which are believed to influence physiological balance and disease states. A “warm and dry” temperament is traditionally associated with stimulation of metabolism, dissolution of accumulated humors, and cleansing effects on bodily tissues. Avicenna notes that turmeric possesses strong cleansing properties and recommends chewing it for the relief of toothache. Its expressed juice was also described as beneficial for ocular conditions, where it was believed to help remove ocular opacities and improve vision. In addition, turmeric was considered a remedy for jaundice [3]. Furthermore, turmeric has been described as possessing strong coloring properties and is traditionally associated with the biliary system and yellow bile [4]. In traditional medicine systems, turmeric has been employed for the management of liver and respiratory conditions, as well as allergic disorders, loss of appetite, and sinusitis [1]. Traditional medical practitioners in historical systems such as Persian and traditional herbal medicine have applied turmeric for joint pain, eye disorders like cataracts, toothaches, and skin infections including herpes and scabies. It has also been recommended for jaundice, heart rhythm issues, and neurological conditions such as paralysis and epilepsy [5]. Furthermore, turmeric, often called “Indian saffron” or “the Golden Spice,” has long been valued in Indian households as a spice, preservative, and traditional remedy [5, 6]. Turmeric has long been used throughout India for many health concerns, much like its applications in traditional Iranian medicine. It serves as a general stomach tonic and is considered helpful for purifying the blood, improving digestion, reducing fever, and supporting the healing of wounds. People also use it to ease nausea during pregnancy and to address various skin and liver ailments. Externally, turmeric is applied for conditions such as arthritis, conjunctivitis, eczema, hemorrhoids, and skin infections. Additionally, many Indian women traditionally spread turmeric on the skin to help slow unwanted hair growth [5, 7, 8]. In addition, turmeric has earned formal recognition in Chinese, Japanese, and Korean pharmacopeias and is used to treat various conditions, including joint inflammation, hepatitis, skin disorders, and wounds [9].

**FIGURE 1:**
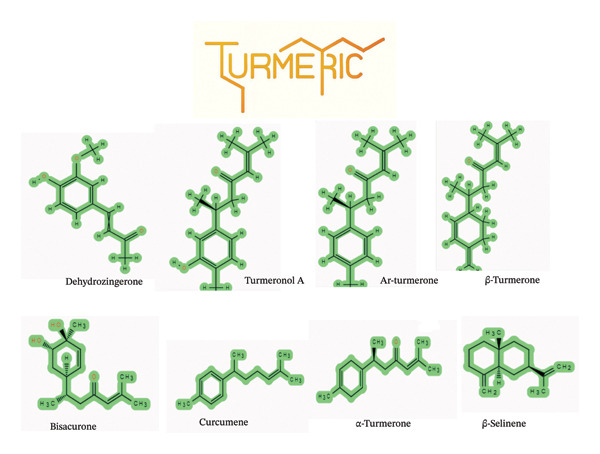
Representative chemical structures of major bioactive compounds found in turmeric.

Phytochemical investigations have identified three major classes of compounds in turmeric: curcuminoids, sesquiterpenes, and a group known as terpecurcuminoids. Among these, curcuminoids represent the principal bioactive constituents. To date, more than 20 individual curcuminoids have been isolated, with bisdemethoxycurcumin, curcumin, and demethoxycurcumin being the most abundant. Further studies have classified approximately 60 sesquiterpenoid compounds in turmeric, predominantly of the bisabolane type. Notable examples include 4,5‐dihydroxybisabola‐2,10‐diene, α‐turmerone, β‐selinene, β‐turmerone, ar‐turmerone, bisacurone, curcumene, dehydrozingerone, and turmeronol A (Figure 1). More recently, a distinct category of minor constituents termed terpecurcuminoids has been described. These molecules are structural hybrids, formed by the conjugation of curcuminoids with either monoterpenes or sesquiterpenes. At least 29 terpecurcuminoids have been documented so far, including 15 derivatives linked via carbon–carbon bonds, 12 via carbon–oxygen bonds, and 2 conjugated with menthane‐type monoterpenoids [10]. The diversity of these phytochemical constituents contributes to the wide range of biological activities attributed to turmeric and is relevant for interpreting both its pharmacological effects and potential toxicity. Importantly, variations in the relative concentrations of these constituents among different turmeric preparations and extracts may influence both biological activity and toxicity profiles [11, 12], highlighting the importance of phytochemical composition when interpreting experimental and clinical safety data.

**FIGURE 2:**
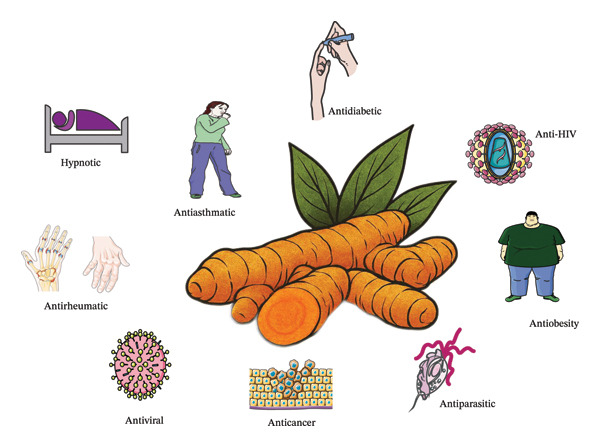
Illustration of turmeric and its proposed biological effects across multiple health domains (images from https://smart.servier.com).

Numerous studies have demonstrated that *C*. *longa* and its active constituents exhibit a wide array of pharmacological effects, including antioxidant, anti‐inflammatory [13], antirheumatic [14], hypnotic [15], antidepressant [16], antiobesity [17], antiasthmatic [18], and antiallergic [19] (Figure 2).

In addition to its historical and pharmacological significance, turmeric has undergone formal safety evaluations by international regulatory bodies. The European Food Safety Authority (EFSA) has assessed various turmeric‐derived preparations, including extracts, oils, oleoresins, and tinctures, for use as sensory additives in animal feed and drinking water. These formulations were considered safe at specified exposure levels across multiple species, with no consumer health concerns identified. However, turmeric additives may act as irritants to the skin, eyes, and respiratory tract and are considered skin sensitizers. Moreover, curcumin (E 100), a purified turmeric extract, is authorized in the European Union as a food colorant. Its safety profile has been reviewed by both EFSA and the Joint Food and Agriculture Organization of the United Nations (FAO)/World Health Organization (WHO) Expert Committee on Food Additives (JECFA), which established an acceptable daily intake of 0–3 mg/kg body weight (WHO, 2004; EFSA ANS Panel, 2010). The European Medicines Agency (EMA) has also recognized *C. longa* rhizome as an herbal medicinal product in various extract forms [20].

Although turmeric and its constituents are generally regarded as safe, isolated reports have raised concerns about potential adverse effects. A small number of clinical cases have linked turmeric supplementation to severe hepatitis [21, 22], highlighting the need for careful assessment of its toxicological boundaries. Additionally, a retrospective case–control study in patients with advanced pancreatic cancer reported that curcumin supplementation was associated with accelerated muscle and fat loss, particularly in sarcopenic individuals, leading to significantly reduced survival compared to untreated controls (median 169 vs. 299 days; *p* = 0.024) [23]. These findings underscore the importance of evaluating the safety of turmeric across vulnerable populations and clinical contexts.

Considering widespread use and its well‐documented pharmacological potential of turmeric, concerns remain regarding its safety profile, particularly in relation to dosage, bioavailability, and long‐term exposure. While curcumin and related compounds are generally considered safe, emerging data on their toxicological thresholds, metabolic interactions, and regulatory limits warrant closer examination. This narrative review aims to critically evaluate the existing evidence on the toxicity and safety of *C*. *longa* and its major constituents, with a focus on both experimental findings and regulatory assessments. By synthesizing data from traditional medicine, phytochemistry, pharmacology, and international safety evaluations, this work seeks to clarify the therapeutic window of turmeric and guide its responsible use in clinical, dietary, and commercial contexts.

### 1.1. Phytochemical Basis of Turmeric Bioactivity and Toxicity

The biological activities and safety profile of *C*. *longa* are closely related to its complex phytochemical composition. The principal active constituents are the curcuminoids, including curcumin, demethoxycurcumin, and bisdemethoxycurcumin, which represent the major polyphenolic pigments of the rhizome [24]. These compounds are widely recognized for their antioxidant, anti‐inflammatory, and anticancer properties [25] and have been the primary focus of pharmacological and toxicological investigations.

In addition to curcuminoids, turmeric contains a variety of volatile oil components, predominantly sesquiterpenes such as monoterpenes [24], α‐turmerone, β‐turmerone, cinol, and zingiberene, sesquiterpenes, d‐sabinene, borneol, and d‐α‐phellandrene [26, 27]. These constituents may influence the pharmacokinetic behavior of curcuminoids, including absorption and metabolism, and can contribute to the overall biological activity of turmeric extracts.

Variations in the relative concentrations of curcuminoids and essential oil components across different turmeric preparations may significantly affect both therapeutic outcomes and toxicity profiles. For example, highly concentrated extracts or formulations designed to enhance curcumin bioavailability may alter systemic exposure and potentially influence dose‐dependent adverse effects [28]. Therefore, understanding the phytochemical composition of turmeric preparations is essential for interpreting experimental toxicity data and for establishing safe therapeutic and dietary exposure levels.

## 2. Methods

A structured literature search was conducted to identify studies evaluating the toxicity and safety of *C. longa* and its bioactive constituents. Four electronic databases, PubMed, Scopus, Web of Science, and Google Scholar, were searched for relevant publications available up to October 2025. No restriction on publication year was applied in order to capture both early foundational studies and recent toxicological investigations.

The search strategy combined keywords and Boolean operators related to turmeric and toxicological outcomes, including: “*Curcuma longa,*” “turmeric,” “curcumin,” “curcuminoids,” AND “toxicity,” “safety,” “adverse effects,” “organ toxicity,” “organ damage,” “genotoxicity,” “mutagenicity,” “carcinogenicity,” “carcinogenesis,” “teratogenicity,” “acute toxicity,” “sub‐acute toxicity,” “chronic toxicity,” “in vivo,” “in vitro,” and “animal study.”

Titles and abstracts were initially screened to identify potentially relevant studies. Full texts of eligible articles were subsequently evaluated for inclusion based on predefined criteria. In addition, the reference lists of relevant articles were manually examined to identify additional studies that may not have been captured during the database search.

This narrative review included evidence from in vitro studies, animal experiments, and clinical investigations to provide a broad overview of the toxicological profile of *C. longa* and its major constituents. However, no clinical studies specifically addressing toxicological endpoints that met the predefined inclusion criteria were identified.

### 2.1. Inclusion Criteria

• Original research articles presenting experimental or clinical data
• Studies evaluating toxicological endpoints of turmeric, curcumin, or related compounds
• Articles published in English

### 2.2. Exclusion Criteria

• Review articles, meta‐analyses, editorials, and commentaries
• Studies lacking toxicological relevance or endpoints
• Non‐English publications

As this study was designed as a narrative review, the aim was to provide a comprehensive synthesis of the available evidence rather than a quantitative systematic analysis. Therefore, a formal PRISMA‐based screening process and exact counts of retrieved and excluded articles were not recorded. In addition to primary research articles, safety evaluations and toxicological reports from international regulatory organizations, such as EFSA and JECFA, were also consulted to provide additional context for the toxicological profile of *C*. *longa* and its main components.

## 3. Toxicity Assessment of Turmeric Based on Experimental Models

An overview of the existing literature concerning the toxicity of turmeric and its main components is provided in the following section. It draws upon multiple studies that have explored this aspect from different angles. In the following sections, toxicity findings are presented separately for crude turmeric preparations (such as powders, decoctions, and solvent extracts) and for purified isolates, including curcumin and other curcuminoids. This distinction is made because crude extracts contain complex mixtures of constituents, whereas studies using isolated compounds allow the attribution of specific toxicological effects to defined molecules.

### 3.1. Pregnancy‐Related Toxicity

#### 3.1.1. In Vitro

##### 3.1.1.1. *C. longa* Main Components

###### 3.1.1.1.1. Curcumin

Mouse blastocysts were exposed to curcumin (6, 12, and 24 μM) to assess cytotoxicity and developmental potential. At the highest concentration (24 μM), curcumin significantly increased apoptosis and reduced total cell number in blastocysts. Despite these cellular effects, in vitro implantation assays using fibronectin‐coated culture dishes showed no significant differences in implantation success between curcumin‐treated and control embryos [29].

Curcumin exposure during in vitro maturation (IVM) of mouse oocytes significantly impaired reproductive outcomes. Treated oocytes exhibited reduced maturation and fertilization rates, along with compromised embryonic development. Postimplantation analysis revealed increased embryo resorption and decreased fetal weight, indicating that curcumin disrupts early developmental processes. Further evaluation showed that these effects were mediated by caspase‐3–dependent apoptosis, as pretreatment with a caspase‐3 inhibitor effectively mitigated curcumin‐induced embryotoxicity [30].

A cell‐based study explored the embryotoxic potential of curcumin using chick cardiomyocytes cultured in micromass. High doses significantly impaired cellular activity and protein content. In contrast, low‐dose treatment showed no adverse impact compared to controls. Remarkably, coadministration of ethanol—a known teratogen—with a nontoxic curcumin dose resulted in additive toxicity [31].

#### 3.1.2. In Vivo

##### 3.1.2.1. *C. longa* Powder or Extracts

The impact of turmeric decoctum was investigated on angiogenic markers, vascular endothelial growth factor (VEGF), and vascular endothelial (VE) cadherin, in chick embryos. The decoctum was prepared via boiling and freeze‐drying and then administered in ovo to embryonated chicken eggs. Following injection into the yolk and a 48‐h incubation period, the expression of VEGF and VE cadherin was assessed. The results indicated that turmeric decoctum, even at the highest tested dose, did not alter the expression levels of these angiogenic molecules [32].

Assessing the embryotoxic and teratogenic potential of the methanolic extract of *C*. *longa* using zebrafish as a vertebrate model revealed that embryos exposed to doses above 62.5 μg/mL exhibited dose‐dependent toxicity, with 125 μg/mL inducing significant mortality and morphological abnormalities in hatched larvae. Observed malformations included bent trunks, kinked tails, and yolk sac edema, indicating pronounced teratogenic effects at higher concentrations [33].

The embryotoxic potential of alcoholic extracts of *C. longa* was assessed in mice. Pregnant females were administered sublethal doses during Gestational Days 7 to 14, while a control group remained untreated. Although no statistically significant changes were observed in embryo length or weight, all treated groups exhibited a marked reduction in maternal body weight. Embryos exposed to higher concentrations displayed multiple structural anomalies, including micromelia, hemorrhagic lesions, cleft lip, and cases of resorption [34].

A preclinical investigation evaluated the potential fertility‐enhancing effects of turmeric supplementation in female rats. Sexually mature rats with regular estrous cycles were randomly assigned to three groups and administered either distilled water (control), two doses of turmeric extract over a two‐week period, including the mating phase. On Gestational Day 20, laparotomy was performed to assess reproductive parameters such as uterine implantation count, corpora lutea, viable offspring, and fetal growth indices. The results revealed no statistically significant differences in any of the measured outcomes between turmeric‐treated groups and controls [35].

##### 3.1.2.2. *C. longa* Main Components

###### 3.1.2.2.1. Curcumin

A two‐generation reproductive toxicity study was conducted in rats to evaluate the safety of dietary curcumin in accordance with Organization for Economic Co‐operation and Development (OECD) Guideline No. 416 and good laboratory practice standards. Curcumin was incorporated into the diet and administered to male and female rats across two successive generations. No treatment‐related adverse effects were observed in parental animals, and reproductive parameters remained unaffected. However, a slight reduction in preweaning body weight gain was noted in F2 offspring at the highest dose. The study established a no observed adverse effect level (NOAEL) of 10,000 ppm, corresponding to daily intakes of approximately 847–1076 mg/kg body weight across sexes and generations. Nonetheless, the JECFA later revised this threshold, citing the F2 weight reduction as a limiting factor, and set an acceptable daily intake of 0–3 mg/kg body weight based on mid‐dose exposures (250–320 mg/kg) considered the no observed effect level (NOEL) [36].

To evaluate postimplantation development, curcumin‐pretreated blastocysts were transferred into the uteri of pseudopregnant mice. Embryos previously exposed to 24 μM curcumin exhibited reduced implantation rates, increased resorption frequencies, and significantly lowered fetal weights compared to controls. These outcomes indicate that while in vitro implantation may appear unaffected, prior curcumin exposure compromises subsequent in vivo development. Mechanistic analysis implicated reactive oxygen species (ROS) generation and mitochondrial apoptotic signaling as key contributors to curcumin‐induced embryotoxicity [29].

In a complementary in vivo model, 21‐day‐old female ICR mice were randomly assigned to control or treatment groups (*n* = 20 per group) and administered a standard diet with drinking water containing curcumin (10–40 μM) for 4 days, while control animals received curcumin‐free water. To obtain *in vivo*–matured oocytes, mice underwent standard superovulation using equine chorionic gonadotrophin followed by human chorionic gonadotrophin, after which mature ova were collected from the oviducts. Curcumin intake resulted in a significant reduction in oocyte maturation rates, accompanied by decreased in vitro fertilization success and impaired early embryonic development compared with controls. Further analyses in the original study indicated that these adverse reproductive outcomes were primarily mediated through caspase‐3–dependent apoptotic pathways, as pretreatment with a caspase‐3–specific inhibitor effectively prevented curcumin‐induced oocyte and embryonic injury [30].

The administration of graded doses of curcumin to pregnant mice during Gestational Days 6 to 15 indicated that the highest dose significantly increased embryo resorption rates and led to dose‐dependent reductions in fetal weight and crown‐rump length. Craniofacial anomalies, including flattened nasal bridge and micrognathia, were observed, alongside skeletal defects such as enlarged anterior fontanelle, misaligned sternal ossification centers, and delayed ossification in limbs and vertebrae. No significant differences were found in the incidence of supernumerary ribs [37].

Evaluating the maternal and fetal safety of curcumin delivered via lipid‐core nanocapsules (C‐LNCs) formulated with poly(ε‐caprolactone), administered orally during the organogenesis phase in pregnant rats illustrated that maternal health indicators, including body weight, clinical signs, and intake patterns, remained unaffected across all groups. On Gestational Day 20, fetal biometric assessments and external anomaly screenings revealed no developmental delays or morphological defects. Histopathological analysis of placental tissue showed no structural alterations. Notably, both curcumin and C‐LNC treatments led to a reduction in placental tumor necrosis factor‐alpha (TNF‐α) levels [38] (Table 1).

**TABLE 1:** Summary of studies assessing pregnancy‐related toxicity of turmeric extracts and curcumin.

Extract/compound	Species	Doses/duration	Results	Ref.
*In vitro*				
Curcumin	Mouse blastocysts	6, 12, 24 μM	‐ No significant differences in implantation success between curcumin‐treated and control embryos ↑ Apoptosis ↓ Total cell numbers	[29]

Curcumin	Mouse oocytes	0, 5, 10, 20 μM	↑ Embryo resorption, caspase‐3–dependent apoptosis ↓ Maturation and fertilization rates, fetal weight	[30]

Curcumin	Chick cardiomyocytes	50, 500 nM, 1–20 μM	↓ Cellular activity, protein content, cytotoxicity	[31]

*In vivo*				
Turmeric decoctum	Embryonated chicken eggs	200, 300, and 400 ppm, 48‐h incubation	‐ No significant effect on VEGF and VE cadherin expression	[32]

Turmeric methanolic extract	Zebrafish fertilized embryos	7.8, 15.63, 31.25, 62.5, 125 μg/mL	↑ Dose‐dependent toxicity, mortality, morphological abnormalities in hatched larvae, kinked tails, bent trunks, yolk sac edema	[33]

Turmeric alcoholic extract	Female pregnant white Swiss mice	10, 50, and 100 mg/kg, 7 days, gavage	‐ No significant effect on embryo length or weight ↑ Embryos structural anomalies, resorption, micromelia, hemorrhagic lesions, cleft lip ↓ Maternal body weight	[34]

Turmeric powder	Female sexually mature Wistar rats	250, 500 mg/kg, 2 weeks, gavage	‐ No significant effect on corpora lutea, fetal growth indices, uterine implantation count, viable offspring	[35]

Curcumin	Wistar rats	1500, 3000, and 10,000 ppm, p.o.	‐ No treatment‐related adverse effects were observed in parental animals and reproductive parameters ↓ Preweaning body weight gain in F2 offspring	[36]

Curcumin	Pseudopregnant ICR mice	6, 12, 24 μM, 24 h	↑ Resorption frequencies, ROS, mitochondrial apoptotic signaling ↓ Implantation rates, fetal weights	[29]

Curcumin	Female ICR mice	10–40 μM, 4 days, p.o.	↓ Oocyte maturation, in vitro fertilization success, early embryonic development	[30]

Curcumin	Pregnant ICR mice	1.05, 1.52, 2.0 mg/g, 10 days, p.o.	‐ No significant differences in the incidence of supernumerary ribs. ↑ Embryo resorption rates, flattened nasal bridge, micrognathia, enlarged anterior fontanelle, misaligned sternal ossification centers, delayed ossification in limbs and vertebrae ↓ Fetal weight and crown‐rump length	[37]

Curcumin and its nanocapsules	Pregnant Wistar rats	2 mg/kg, 7 days, p.o.	‐ No significant effect on maternal health indicators, no developmental delays, or morphological defects ↓ Placental TNF‐α levels	[38]

Abbreviations: p.o., per os (by mouth); Ref, reference; ROS, reactive oxygen species; TNF‐α, tumor necrosis factor‐alpha; VE cadherin, vascular endothelial cadherin; VEGF, vascular endothelial growth factor.

**FIGURE 3:**
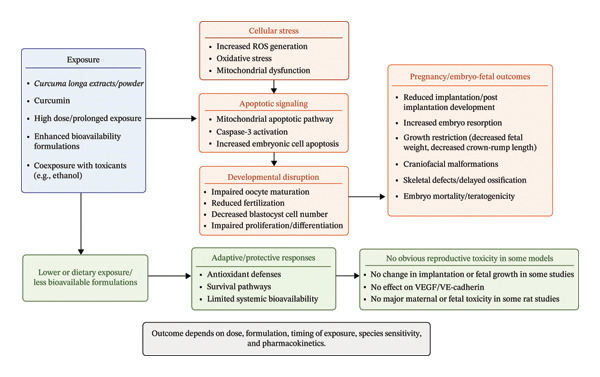
Pregnancy‐related toxicity of *Curcuma longa* and curcumin: proposed mechanistic pathway.

The collective findings from in vitro and in vivo studies evaluating pregnancy‐related toxicity of *C. longa* and its main component curcumin suggest a complex and dose‐dependent toxicological profile (Figure 3). Several experimental models indicate that exposure to curcumin during critical stages of embryogenesis can adversely affect reproductive and developmental outcomes. In vitro studies have demonstrated that curcumin exposure increases apoptosis in mouse blastocysts and reduces total cell numbers [29], while also impairing oocyte maturation, fertilization, and early embryonic development through caspase‐3–dependent apoptotic pathways [30]. Similar embryotoxic responses have been observed in micromass cultures of chick cardiomyocytes, where high concentrations of curcumin significantly reduced cellular activity and protein synthesis [31]. These observations collectively suggest that curcumin can interfere with cellular proliferation and differentiation processes that are essential for early embryonic development.

Findings from these in vitro models indicate that curcumin‐induced cellular stress is frequently associated with increased ROS generation, mitochondrial dysfunction, and activation of caspase‐dependent apoptotic pathways. Together, these processes provide a reasonable biological explanation linking curcumin exposure to impaired embryonic cell survival and reduced developmental competence. However, it is important to note that these mechanistic observations are model‐specific and have not been consistently examined across studies, which limits the development of an integrated mechanistic framework.

**FIGURE 4:**
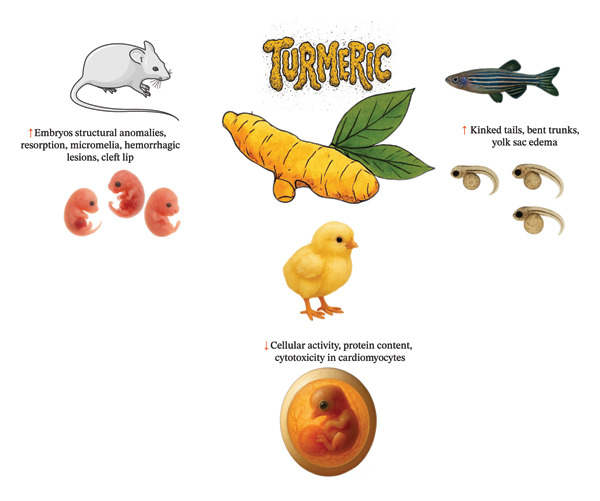
The effects of turmeric and its main bioactive components on teratology and embryonic developmental deficits (images from https://smart.servier.com).

Evidence from in vivo models further supports the dose‐sensitive nature of these effects. Zebrafish embryos exposed to higher concentrations of *C. longa* extracts exhibited mortality and morphological abnormalities, including bent trunks and yolk sac edema, indicating teratogenic potential at elevated exposure levels [33] (Figure 4). Likewise, the administration of alcoholic extracts of *C. longa* to pregnant mice during organogenesis produced structural abnormalities such as micromelia, hemorrhagic lesions, and cleft lip in developing embryos [34]. Curcumin‐specific studies in mice have similarly reported increased embryo resorption and dose‐dependent reductions in fetal weight and crown‐rump length, accompanied by craniofacial and skeletal malformations at higher doses [37]. Although these findings align with oxidative stress‐ and apoptosis‐associated outcomes observed in vitro, direct experimental evaluation of upstream regulators (e.g., p53‐related pathways and DNA damage responses) is largely lacking in reproductive toxicity models, limiting mechanistic integration across systems.

Mechanistic investigations provide direct evidence that curcumin induces embryotoxicity through oxidative stress and apoptosis‐related pathways, as demonstrated by elevated intracellular ROS levels and mitochondrial dysfunction in mouse blastocysts [29], as well as caspase‐3–dependent apoptotic activation observed in both in vitro and in vivo mouse models, where caspase‐3 inhibition preserved oocyte maturation and early embryonic development [30]. Taken together, these data support a mechanistic framework in which curcumin‐induced ROS generation triggers mitochondrial perturbation and activation of caspase‐dependent apoptotic pathway, ultimately leading to implantation failure, growth restriction, and structural malformations under conditions of high or prolonged exposure. Nonetheless, the available evidence does not yet clarify whether these pathways operate similarly across different species, formulations, or exposure windows, indicating the need for more harmonized mechanistic studies.

In contrast, several studies suggest that turmeric or curcumin may not exert obvious reproductive toxicity under certain experimental conditions. For example, turmeric decoctum administered to chick embryos did not significantly alter the expression of angiogenic markers such as VEGF and VE cadherin [32], and turmeric supplementation in female rats did not affect implantation rates, fetal growth indices, or other reproductive parameters [35]. Moreover, a two‐generation reproductive toxicity study conducted in accordance with OECD Guideline No. 416 reported no treatment‐related adverse effects on reproductive performance or offspring development in rats exposed to dietary curcumin, although a slight reduction in preweaning body weight in F2 offspring at the highest dose was noted [36]. Similarly, oral administration of C‐LNCs during rat organogenesis did not produce maternal or fetal toxicity, while reducing placental TNF‐α levels [38]. These findings indicate that, when exposures remain within ranges consistent with dietary intake or clinically relevant dosing, endogenous antioxidant defenses and adaptive signaling pathways may be sufficient to moderate curcumin‐induced oxidative stress, preventing progression to apoptosis and teratogenicity. This contrast highlights the importance of dose, formulation, and timing of exposure when interpreting toxicological outcomes.

The apparent discrepancies among these findings likely reflect differences in experimental design, species sensitivity, dosage levels, and formulation characteristics. In vitro systems often employ concentrations that exceed physiologically achievable levels following oral intake, which may exaggerate cytotoxic effects. Conversely, whole‐animal studies incorporate metabolic processes and pharmacokinetic factors that can modulate systemic exposure and toxicity. Variability in extraction methods and formulations of *C. longa* may also influence the bioavailability and biological activity of curcumin and related curcuminoids, potentially explaining divergent outcomes across models [33–38]. Additionally, some studies demonstrated that curcumin may interact with other toxicants, as evidenced by the additive embryotoxicity observed when curcumin was coadministered with ethanol in cell‐based assays [31].

These interactions suggest that curcumin may enhance pre‐existing oxidative or inflammatory stress, thereby reducing the threshold required to trigger mitochondrial dysfunction and apoptosis‐related pathways.

A critical comparison of in vitro and in vivo data also highlights important differences in dose relevance and biological context. In vitro assays typically expose embryos or isolated cells directly to micromolar concentrations of curcumin, in the absence of physiological barriers, binding proteins, or metabolic clearance, which can accentuate ROS production and apoptotic signaling. In vivo, however, oral administration leads to extensive metabolism, limited systemic bioavailability, and distribution across maternal and fetal compartments, resulting in substantially lower free curcumin concentrations at the target sites.

Consequently, marked activation of oxidative stress‐related apoptotic pathways sufficient to induce malformations or pregnancy loss appears to occur mainly under conditions of high experimental dosing or when formulations significantly increase curcumin bioavailability, whereas typical dietary exposure is unlikely to reach such levels. Therefore, dose relevance remains a key consideration when generalizing preclinical findings to human pregnancy.

Taken together, the current body of evidence suggests that while turmeric and curcumin are generally considered safe at dietary levels, exposure to high concentrations or pharmacological formulations during pregnancy may pose risks to embryonic development. These risks appear to be mediated primarily through oxidative stress and apoptosis‐related mechanisms that disrupt early cellular differentiation and implantation processes [29, 30]. Integration of findings across models indicates that the balance between pro‐oxidant/apoptotic signaling and protective responses (e.g., antioxidant defenses and survival pathways) is a key determinant of outcome and that this balance is strongly influenced by dose, timing of exposure, and formulation‐dependent pharmacokinetics. At the same time, the absence of reproductive toxicity in several animal studies and regulatory assessments indicates that the safety profile of curcumin is strongly influenced by dose, formulation, and exposure duration [36, 38].

Despite the valuable insights provided by preclinical research, several limitations constrain the translation of these findings to human pregnancy. Most available evidence derives from animal models or in vitro systems, which may not fully replicate human placental physiology or maternal–fetal pharmacokinetics. In addition, studies vary considerably in dosing strategies, gestational timing, and outcome measures, making direct comparisons difficult. Human clinical data evaluating the reproductive safety of turmeric or curcumin supplementation during pregnancy remain limited. Moreover, relatively few studies have systematically examined upstream regulatory pathways, such as p53 activation, DNA damage signaling, or detailed mitochondrial bioenergetics, which limits a clear understanding of the mechanistic sequence linking exposure to adverse outcomes. Future research should therefore prioritize well‐designed reproductive toxicity studies using standardized formulations and clinically relevant exposure levels. Investigations exploring pharmacokinetic profiles, placental transfer, and long‐term developmental outcomes will also be essential to establish evidence‐based safety recommendations. Integrative studies combining oxidative stress markers, apoptosis regulators, and pathway‐level analyses across both in vitro and in vivo systems would be particularly valuable for clarifying the mechanistic basis of curcumin‐associated developmental toxicity.

Generally, the available evidence highlights the importance of cautious use of turmeric‐derived supplements during pregnancy. While dietary consumption of turmeric as a culinary spice is unlikely to reach levels associated with developmental toxicity, high‐dose supplementation or concentrated curcumin formulations may warrant careful evaluation until more robust human safety data become available. Clinicians and pregnant individuals should therefore consider both dose and formulation when assessing risk, recognizing that mechanistic data point to a potential hazard at high exposures, even in the absence of definitive human epidemiological evidence.

### 3.2. Mutagenic Activity and Cancer Risk

#### 3.2.1. In Vitro

##### 3.2.1.1. *C. longa* Oil

The mutagenic potential of turmeric essential oil was assessed using the Ames test across four *Salmonella typhimurium* strains: TA98, TA100, TA102, and TA1535. These strains were tested both with and without metabolic activation. Turmeric essential oil did not induce mutations in any of the strains under either condition, indicating a lack of mutagenicity in bacterial cells exposed to the essential oil [39].

##### 3.2.1.2. *C. longa* Main Components

###### 3.2.1.2.1. Curcumin

Examining the mutagenic potential of silver nanoparticles synthesized using curcumin via the Salmonella/microsome assay (Ames test) disclosed that in the absence of metabolic activation, curcumin nanoparticles did not exhibit mutagenicity. However, under metabolic activation conditions, curcumin nanoparticles can act as indirect mutagens following enzymatic biotransformation [28] (Table 2).

**TABLE 2:** Summary of studies assessing mutagenic and genotoxic potential of turmeric and curcumin.

Extract/compound	Species	Doses/duration	Results	Ref.
*Mutagenic activity and cancer risk*				
In vitro				
Turmeric essential oil	*S. Typhimurium* strains	100, 1000, 3000 μg/plate	‐ No mutations in any of the strains under either condition	[39]

Silver nanoparticles synthesized using curcumin	*S. Typhimurium* strains	0.0010–0.0081 mg/plate	‐ No mutagenicity in the absence of metabolic activation ‐ Mutagenic responses were observed under metabolic activation	[28]

Curcumin (Longvida®)	*S. Typhimurium* strains	39.1, 78.1, 156.3, 312.5, 625, 1250, 2500, 5000 μg/plate	‐ No mutagenic changes in any strain	[40]

*Genotoxic potential*				
In vitro				
Turmeric crude and ethanolic extracts	Human genomic DNA	5, 10, 25, 50, 100, 200 mg/mL	Crude extract: ↑ DNA fragmentation Ethanolic extract: ‐ No DNA damage	[41]

Curcumin	PC12 cells	0.5–128 μg/mL	↑ Cytotoxicity, micronuclei formation	[11]

Curcumin	Human peripheral lymphocytes	0–50 μg/mL	↑ Chromosome aberrations predominantly acentric fragments, mitotic index ↓ Proliferation index	[42]

Curcumin	HT1080 human cells with wild‐type p53	0.06–100 µM	↑ DNA‐damage response activity, ROS, phospho‐H2AX levels, p53 induction, G2/M cell cycle arrest, apoptosis, micronuclei formation	[43]

Curcumin solid dispersions	*A. cepa* root tip cells	—	‐ No chromosomal abnormalities or other genotoxic effects	[44]

Curcumin	*S. mansoni*	5–100 μg/mL	↑ Antischistosomal activity against adult *S. mansoni*, morphological disruption	[45]

In vivo				
Turmeric essential oil	Wistar rats	1 g/kg, 14 days	‐ No genotoxic effect	[39]

Curcumin (Longvida®)	Swiss albino mice	500, 1000, 2000 mg/kg, 2 days, p.o.	‐ No structural or numerical chromosomal damage in somatic cells	[40]

Abbreviations: DNA, deoxyribonucleic acid; phospho‐H2AX, phosphorylated H2A histone family member X; p.o., per os (by mouth); Ref, reference; ROS, reactive oxygen species.

Longvida, a solid lipid formulation of curcumin designed to enhance bioavailability, was evaluated using the bacterial reverse mutation assay. The test employed multiple *S. typhimurium* strains to detect base‐pair and frame‐shift mutations at the histidine locus. Longvida did not induce mutagenic changes in any strain, regardless of the presence or absence of metabolic activation [40].

##### 3.2.1.3. Multicompound Studies

An in silico toxicity screening was conducted on 200 chemical constituents derived from *C. longa* to identify compounds with favorable safety profiles. The assessment included computational predictions of bacterial mutagenicity, rodent carcinogenicity, and human hepatotoxicity. Among the tested compounds, 184 were flagged as toxic, with 136 showing mutagenic potential, 153 predicted as carcinogenic, and 64 identified as hepatotoxic. Curcumin and its derivatives were among those associated with dose‐dependent hepatotoxicity. In contrast, a subset of turmeric compounds demonstrated no mutagenic, carcinogenic, or hepatotoxic effects, suggesting that certain constituents may offer safer therapeutic potential [46].

**FIGURE 5:**
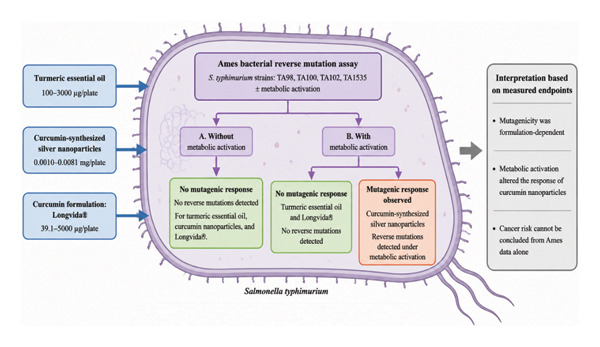
Mutagenic activity and cancer risk of *Curcuma longa*‐derived preparation: measured in vitro endpoints.

The available evidence evaluating the mutagenic and carcinogenic potential of *C. longa*–derived compounds suggests a heterogeneous safety profile that depends strongly on chemical composition, formulation, and metabolic conditions (Figure 5). Experimental studies generally indicate that certain turmeric preparations exhibit minimal mutagenic activity under standard testing conditions. For instance, turmeric essential oil showed no mutagenic effects in multiple *S*. *typhimurium* strains in the Ames test, regardless of metabolic activation status, suggesting that the volatile constituents of *C. longa* may lack direct DNA‐damaging potential in bacterial systems [39]. Similarly, Longvida, a lipid‐based curcumin formulation developed to enhance bioavailability, did not induce reverse mutations in several *S. typhimurium* strains in bacterial mutagenicity assays, indicating that this formulation does not exhibit mutagenic properties under the tested experimental conditions [40]. These results collectively indicate that formulations lacking reactive metabolites under assay conditions tend to show low mutagenic activity in bacterial systems.

In contrast, some curcumin‐derived formulations demonstrate conditional mutagenic behavior depending on metabolic activation. Silver nanoparticles synthesized using curcumin were nonmutagenic in the absence of metabolic activation but exhibited mutagenic activity when enzymatic metabolic systems were present, suggesting that metabolic transformation may produce reactive intermediates that can bind to nucleophilic sites in DNA or proteins [28]. Such reactive metabolites may form through oxidative or conjugative metabolic pathways that modify the chemical structure of curcumin, reflecting its ability to act as an antioxidant under some conditions and a pro‐oxidant under others. This conditional response highlights metabolic activation as a key mechanistic determinant of mutagenicity, providing a unifying explanation for discrepancies observed across curcumin formulations. These findings highlight the importance of metabolic context and redox balance in toxicological assessment, as metabolic enzymes may convert otherwise inert compounds into reactive species with genotoxic potential.

The complexity of mutagenicity within *C. longa* phytochemistry is further illustrated by large‐scale compound screening approaches. A computational toxicology screening of 200 compounds derived from turmeric predicted mutagenic potential for a considerable number of constituents, with several also identified as having possible carcinogenic and hepatotoxic properties [46]. Although these predictions require experimental validation, they emphasize that turmeric contains chemically diverse constituents with distinct toxicological profiles. Notably, the same analysis identified several compounds lacking predicted mutagenic or carcinogenic properties, indicating that the safety of turmeric preparations may depend on selective enrichment or removal of specific phytochemicals during extraction or formulation [46]. These in silico findings support the idea that structural diversity within turmeric constituents may contribute to the formulation‐dependent genotoxic responses observed experimentally, although validation in biological systems is still required.

Although curcumin is well known for its extremely low oral bioavailability, this characteristic does not preclude hepatotoxic risk; rather, several studies suggest that poor systemic absorption may shift the metabolic burden toward the liver. Because only a small fraction of unmetabolized curcumin reaches the circulation, the compound undergoes extensive first‐pass metabolism, generating glucuronidated, sulfated, and reductive metabolites within hepatocytes. Under high‐dose or repeated‐dose conditions, this intensified metabolic processing may lead to transient hepatocellular stress, accumulation of reactive intermediates, or compensatory upregulation of detoxification pathways, which aligns with predictions from in silico analyses identifying curcumin and some derivatives as potentially hepatotoxic at elevated exposures [46].

Taken together, the current evidence suggests that mutagenic risk associated with turmeric and its derivatives is not uniform but rather influenced by factors such as molecular structure, metabolic activation, and delivery system. While conventional turmeric oil and certain lipid‐based curcumin formulations appear nonmutagenic in bacterial assays [39, 40], modified forms such as nanoparticle‐based preparations may exhibit altered toxicological behavior due to enhanced cellular uptake or metabolic transformation [28]. Generally, metabolic activation appears to be the key factor that helps explain the differing mutagenic outcomes across models; however, without supporting in vivo data, it remains uncertain whether these same pathways function similarly in mammalian systems. These findings underscore the importance of formulation‐specific safety evaluations when developing turmeric‐derived therapeutics.

Nevertheless, the interpretation of the available data is limited by several methodological considerations. Most studies rely on in vitro bacterial mutagenicity assays, which are valuable screening tools but do not fully replicate mammalian metabolism, DNA repair mechanisms, or long‐term carcinogenic processes. Moreover, computational toxicity predictions may overestimate mutagenic risk because they rely primarily on structural alerts rather than experimental biological outcomes [46]. The lack of in vivo genotoxicity or carcinogenicity studies for many *C. longa* constituents prevents establishing whether the mutagenic responses observed in bacterial systems translate to meaningful cancer risk in mammals. The absence of extensive in vivo genotoxicity studies or long‐term carcinogenicity assessments for many turmeric constituents further limits the ability to extrapolate these findings to human exposure scenarios.

To strengthen mechanistic understanding and relevance, future research should prioritize integrated genotoxicity testing strategies that combine bacterial assays, mammalian cell systems, and in vivo models. In addition, formulation‐specific pharmacokinetic studies and long‐term safety evaluations will be critical to determine whether enhanced bioavailability technologies alter the mutagenic or carcinogenic potential of curcumin‐based products. Studies that examine how different formulations undergo metabolic activation and how these processes relate to DNA reactivity in mammalian systems would provide important perceptions for building a clearer mechanistic understanding. Briefly, the current body of evidence indicates that while many turmeric preparations demonstrate low mutagenic activity in standard assays, the toxicological profile of *C. longa* derivatives remains formulation‐dependent. Careful characterization of individual compounds and delivery systems will therefore be essential to ensure genomic safety as turmeric‐derived products continue to be developed for therapeutic applications.

### 3.3. Genotoxic Potential

#### 3.3.1. In Vitro

##### 3.3.1.1. *C. longa* Extracts

A study was carried out to investigate the in vitro genotoxic properties of crude (unfractionated extracts obtained by solvent extraction without purification or isolation of individual constituents) and ethanolic extracts derived from *C*. *longa* rhizome. The crude extract induced substantial DNA fragmentation (40%–95%) at concentrations of 50–100 mg/mL, exceeding 95% at 200 mg/mL. In contrast, the ethanolic extract did not produce DNA damage at any tested concentration [41].

##### 3.3.1.2. *C. longa* Main Components

###### 3.3.1.2.1. Curcumin

Exploring the cytotoxic and genotoxic/antigenotoxic effects of curcumin in a neuronal model using PC12 cells exposed to cisplatin revealed that at higher concentrations, curcumin alone exhibited cytotoxicity and increased micronuclei formation [11].

An in vitro study assessed the cytogenetic effects of curcumin on human peripheral lymphocytes. At higher concentrations, curcumin induced a significant increase in chromosome aberrations, predominantly acentric fragments, while sister chromatid exchange rates remained statistically unchanged across all treatment groups. Curcumin also altered cell proliferation dynamics, with a significant reduction in proliferation index observed at 2 and 5 μg/mL and a general trend toward increased mitotic index across all tested concentrations [42].

Curcumin demonstrated pronounced DNA‐damage response activity in HT1080 human cells with wild‐type p53. At low micromolar concentrations (LOELs: 3–8 μM), curcumin significantly increased ROS, p53 induction, and phosphorylated H2A histone family member X (phospho‐H2AX) levels, markers of DNA damage, and cellular stress. Curcumin triggered a robust G2/M cell cycle arrest and induced apoptosis. It also elevated micronuclei formation [43].

The genotoxic potential of curcumin solid dispersions was evaluated using the *Allium cepa* assay. The results indicated that curcumin solid dispersions did not induce chromosomal abnormalities or other genotoxic effects in this plant‐based model [44].

Curcumin demonstrated notable antischistosomal activity against adult *Schistosoma mansoni* worms in vitro. A 24‐h exposure to curcumin resulted in a lethal concentration 50 (LC_50_) of 87.25 μg/mL, with a maximum mortality rate of 91.3% observed at 100 μg/mL. Ultrastructural analysis revealed sex‐dependent surface damage to the worms, indicating curcumin‐induced morphological disruption. Additionally, DNA fragmentation assays confirmed the genotoxic effect of curcumin on *S. mansoni* [45].

#### 3.3.2. In Vivo

##### 3.3.2.1. *C. longa* Oil

To evaluate genotoxicity in a mammalian system, Wistar rats were administered turmeric essential oil orally at a dose of 1 g/kg body weight for 14 days. Genotoxic endpoints—including chromosome aberration, micronucleus formation in bone marrow cells, and DNA damage via comet assay—were all negative. These results confirm that turmeric essential oil does not exert genotoxic or carcinogenic effects in vivo under the tested conditions [39].

##### 3.3.2.2. *C. longa* Main Components

###### 3.3.2.2.1. Curcumin

To assess chromosomal integrity in vivo, Longvida was administered orally to mice. The mammalian erythrocyte micronucleus test revealed no evidence of structural or numerical chromosomal damage in somatic cells [40] (Table 2).

The available evidence indicates that the genotoxic profile of *C. longa* and its constituents is highly dependent on extract composition, dose, formulation, and biological context. In vitro studies consistently show that crude or unfractionated *C. longa* extracts can induce substantial DNA damage at high concentrations, as demonstrated by marked DNA fragmentation following exposure to crude rhizome extracts, while more refined ethanolic extracts lacked genotoxic activity under comparable conditions [41]. These differences suggest that the presence or absence of specific phytochemical mixtures, not just curcumin, may influence the initiation of DNA damage in vitro, although the exact constituents responsible have not been fully identified.

Similarly, curcumin exhibited concentration‐dependent genotoxic effects across multiple in vitro models. In neuronal PC12 cells, high concentrations of curcumin increased cytotoxicity and micronucleus formation [11], while studies in human peripheral lymphocytes revealed elevated chromosomal aberrations, primarily acentric fragments, along with altered proliferation indices at higher doses [42]. Mechanistic insights were further supported by findings in HT1080 human cells, where curcumin induced ROS generation, p53 activation, phosphorylation of H2AX, G2/M cell cycle arrest, and apoptosis, all hallmark responses to DNA damage [43]. While these findings collectively point toward oxidative stress‐associated DNA damage signaling, the available studies do not establish a unified mechanistic pathway, partly because each model uses different concentrations, exposure durations, and cellular systems. Therefore, the mechanistic interpretation remains fragmented and context‐dependent.

**FIGURE 6:**
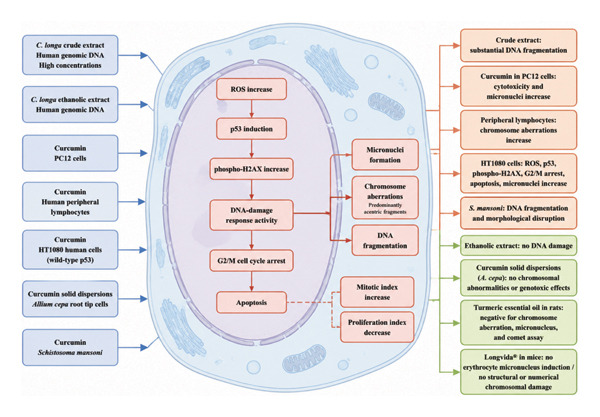
Genotoxic potential of *Curcuma longa*–derived preparation: measured endpoints across models.

Remarkably, some systems do not reproduce these genotoxic effects. The absence of chromosomal abnormalities in the *A. cepa* assay [44] (Figure 6) and the selective DNA fragmentation observed in *S. mansoni* [45] highlight that the biological model strongly shapes the observed response, making it unlikely that a single mechanistic explanation can account for all reported outcomes. Moreover, genotoxic effects in parasites cannot be extrapolated to mammalian toxicity.

In contrast to in vitro findings, in vivo studies in mammals consistently show a lack of genotoxicity. Oral administration of turmeric essential oil to Wistar rats did not induce chromosome aberrations, micronuclei formation, or DNA strand breaks in bone marrow cells, supporting the absence of genotoxic or carcinogenic effects under the tested conditions [39]. Likewise, oral exposure to the lipid‐based curcumin formulation Longvida failed to produce micronuclei in murine erythrocytes, indicating preserved chromosomal integrity in vivo [40]. This discrepancy likely reflects the differences in achievable systemic concentrations, metabolic processing, and bioavailability constraints that limit exposure of mammalian tissues to the high concentrations used in vitro. However, because most in vivo studies assess acute or short‐term exposure, it remains unclear whether similar outcomes would persist under chronic or high‐dose supplementation.

The major strength of the current evidence lies in the use of multiple genotoxicity endpoints across diverse models, allowing mechanistic insights into dose‐dependent effects, yet several limitations constrain mechanistic integration. Many in vitro assays rely on supraphysiological concentrations that do not reflect dietary or supplemental exposure levels, contributing to difficulty in forming a dose‐relevant mechanistic framework. Additionally, the heterogeneity of assay conditions and lack of metabolic activation systems reduce the ability to meaningfully compare findings across studies. As a result, the available data do not yet provide a cohesive mechanistic model explaining how genotoxicity arises across different systems.

From a translational perspective, the collective data suggest that turmeric‐derived products, particularly standardized extracts and optimized curcumin formulations, are unlikely to pose significant genotoxic risk when used at physiologically relevant doses. Nevertheless, the considerable variability between different extract types, together with the limited number of long‐term in vivo investigations, suggests that high‐dose preparations and poorly characterized products should be approached with caution and examined more thoroughly. Future studies would benefit from the use of harmonized testing protocols, the inclusion of metabolically competent models, and mechanistic designs that allow a more direct comparison of dose relevance between in vitro systems and in vivo exposures.

### 3.4. Acute to Chronic Toxicity Spectrum

#### 3.4.1. In Vivo

##### 3.4.1.1. *C. longa* Extracts or Oil

Subchronic oral toxicity of turmeric and its ethanolic extract was evaluated in female Swiss mice and Wistar rats over 14‐ and 90‐day dietary exposure periods. Animals received turmeric at 1% and 5% or ethanolic extract at 0.05% and 0.25% concentrations. Prolonged administration of turmeric at 5% for 90 days resulted in significant reductions in body weight gain, altered liver weight indices, and histopathological evidence of hepatotoxicity, specifically focal necrosis and regenerative changes, in both species. Notably, mice exhibited hepatic damage even at lower doses (0.2%–1%) within just 14 days [12].

A 6‐month chronic toxicity study investigated the safety profile of curcuminoids extracted from *C. longa* rhizome in Wistar rats. Animals received daily oral doses of 10, 50, or 250 mg/kg body weight, representing 1×, 5×, and 25× the proposed therapeutic dose, respectively. No significant dose‐dependent changes were observed in hematological parameters across treatment groups. At 50 mg/kg, male rats exhibited a statistically significant increase in the growth rate compared to controls. At the highest dose (250 mg/kg), male rats showed elevated absolute and relative liver weights and increased alkaline phosphatase (ALP) levels, although ALP remained within normal physiological limits. Mild liver and adrenocortical fatty degeneration were noted but did not differ significantly from control groups [47].

In an acute oral toxicity study using Wistar rats, polysaccharide extract of *C. longa* rhizome (NR‐INF‐02, commercially known as Turmacin) was found to be safe at doses up to 5 g/kg body weight. No adverse clinical signs or mortality were observed, indicating a high maximum tolerable dose [48].

Turmeric essential oil was evaluated for oral toxicity in Wistar rats. Acute administration at doses up to 5 g/kg body weight produced no mortality or clinical signs of toxicity. In a 13‐week subchronic study, daily oral doses of 0.1, 0.25, and 0.5 g/kg body weight did not affect body weight, food and water intake, or induce any adverse clinical symptoms. Lipid profile, liver function markers (ALP, alanine aminotransferase [ALT], aspartate aminotransferase [AST]), renal parameters, serum electrolytes, and tissue histopathology remained unchanged across all treatment groups [39].

A 90‐day subchronic oral toxicity study assessed the safety of NR‐INF‐02 in albino Wistar rats. Administered daily at doses of 250, 500, and 1000 mg/kg body weight, NR‐INF‐02 did not produce any mortality or observable clinical signs of toxicity. Parameters including body weight gain, food intake, hematology, hormone levels, ocular and neurological function, serum biochemistry, and urinalysis remained within normal limits across all treatment groups. Organ weight assessments and histopathological examinations revealed no treatment‐related abnormalities. Based on these findings, the NOAEL was established at 1000 mg/kg body weight [49].

Methanolic leaf extract of *C. longa* was evaluated for its phytochemical composition, antioxidant activity, and acute oral toxicity in Wistar rats. Acute toxicity testing at doses ranging from 100 to 2000 mg/kg body weight revealed no signs of intoxication or mortality [50].

A subchronic toxicity study evaluated the safety of repeated oral administration of *C*. *longa* rhizome extract in Wistar rats over a 28‐day period. Rats received turmeric extract at doses of 50, 100, and 200 mg/kg body weight, with no observed adverse effects on liver or kidney biochemical parameters. Hematological profiles remained within normal ranges, indicating no signs of anemia or systemic toxicity. Histopathological examination of major organs revealed no structural abnormalities [51].

In acute oral toxicity testing, a single gavage dose of CURCUGEN, an oleoresin‐based turmeric extract, in Sprague‐Dawley rats yielded a lethal dose 50% (LD_50_) greater than 5000 mg/kg body weight, indicating low acute toxicity. Subchronic administration over 90 days at doses up to 2000 mg/kg/day revealed no treatment‐related adverse effects, with normal clinical, biochemical, and histopathological parameters. The NOAEL was established at 2000 mg/kg/day [52].

Using Lorke’s method, the ethanol extract of *C. longa* was evaluated for acute toxicity via oral and intraperitoneal routes in rats. The calculated LD_50_ values were 3807 mg/kg for oral administration and 774 mg/kg for the intraperitoneal route. Biochemical impact following subacute exposure oral administration of the ethanolic turmeric extract at doses of 400 and 600 mg/kg body weight for 21 days significantly reduced serum total protein and albumin levels, while serum urea levels increased at both doses. Cholesterol levels remained unaffected [53].

##### 3.4.1.2. *C. longa* Main Components

###### 3.4.1.2.1. Curcumin

A solid lipid curcumin particle formulation was evaluated for acute oral toxicity in rodents. The LD_50_ value was found to exceed 2000 mg/kg body weight in both rats and mice. In a 90‐day subchronic study, Wistar rats received daily oral doses of solid lipid curcumin particle at 180, 360, and 720 mg/kg body weight. Across all dose groups, no treatment‐related adverse effects were observed in body weight progression, clinical behavior, feed intake, ophthalmic health, or organ weights. Hematological, biochemical, and urinalysis parameters remained within normal limits, and necropsy revealed no pathological abnormalities. The NOAEL was established at 720 mg/kg/day, the highest dose tested [54].

A modified solid dispersion of curcumin‐loaded nanocomplexes formulated in gums was evaluated for its acute and chronic toxicity in mice and hamsters. Curcumin‐loaded nanocomplexes were administered orally at escalating doses, with acute toxicity assessed up to 11.0 g/kg in mice and 21.4 g/kg in hamsters, and chronic exposure maintained daily for 6 months at doses up to 0.8 g/kg/day in mice and 1.61 g/kg/day in hamsters. Low and medium doses were well tolerated across both species, showing no adverse clinical signs or biochemical alterations. At the highest acute doses, mice exhibited increased spleen‐to‐body weight ratios and elevated biochemical markers, while hamsters showed organ weight changes without biochemical disruption. Chronic high‐dose exposure led to organ weight increases in the gastrointestinal tract of mice and cardiopulmonary tissues of hamsters, accompanied by transient elevations in liver enzymes, glucose, and protein levels. These effects resolved within 28 days post‐treatment. The oral is LD_50_ values were 8.9 g/kg in mice and 16.8 g/kg in hamsters, and NOAELs were established at 0.27 and 0.54 g/kg/day, respectively [55].

Activated curcumin C3 complex (AC3) underwent a comprehensive toxicological evaluation in rodents, including acute, subacute, subchronic, reproductive/developmental, and genotoxicity assessments, all conducted in accordance with OECD good laboratory practice guidelines. In the acute oral toxicity study, a single dose administered to female Wistar rats produced no adverse effects during the 14‐day observation period. Repeated‐dose studies at 125, 250, and 500 mg/kg body weight revealed no treatment‐related clinical, behavioral, reproductive, or developmental abnormalities. Body weight and biochemical and hematological parameters, as well as gross and histopathological findings, remained within normal limits [56].

###### 3.4.1.2.2. Curcuminoids

The curcuminoid‐essential oil complex, a bioavailable turmeric formulation designed to overcome limited gastrointestinal absorption of curcumin, was evaluated for safety in rodents following OECD guidelines. Acute oral administration at a dose of 5000 mg/kg body weight in rats and mice produced no clinical signs of toxicity or mortality. In a 90‐day subchronic study, daily oral dosing of 1000 mg/kg in Wistar rats did not result in any observable adverse effects, with treated animals showing comparable physiological and biochemical parameters to controls [57].

Bisdemethoxycurcumin, a curcuminoid component of *C*. *longa*, was evaluated for its safety profile in rodents through a series of good laboratory practice–compliant studies aligned with OECD guidelines. General toxicity studies conducted over 4 weeks (subacute) and 3 months (subchronic) revealed no treatment‐related adverse effects, establishing a NOAEL of 1000 mg/kg/day. Reproductive and developmental toxicity assessments, including fertility, embryo‐fetal development, and postnatal outcomes, also showed no abnormalities at the same dose [58].

###### 3.4.1.2.3. Tetrahydrocurcuminoids

Tetrahydrocurcuminoids, chemically hydrogenated derivatives of curcuminoids extracted from *C. longa* rhizomes, were assessed by EFSA as a novel food under EU Regulation 2015/2283. Comprising over 95% tetrahydrocurcuminoids, the novel food was proposed for use in food supplements at a dose of 300 mg/day for adults, excluding pregnant and lactating women. Toxicological evaluations, including a 90‐day oral study and a reproduction/developmental toxicity screening, revealed no genotoxic concerns. Based on these findings, EFSA established a safe intake level of 2 mg/kg body weight/day, equivalent to 140 mg/day for the target population. The panel concluded that the novel food is safe at this intake level [59].

Generally, the available in vivo evidence suggests that *C. longa* extracts and their principal constituents exhibit a relatively wide safety margin across acute, subchronic, and chronic toxicity models, although dose‐dependent effects on hepatic parameters have occasionally been reported. Most studies demonstrated low acute toxicity, with LD_50_ values exceeding 2000–5000 mg/kg body weight for turmeric extracts, essential oil, and several standardized formulations in rodents [39, 48, 52]. Similarly, methanolic leaf extracts and polysaccharide‐based preparations showed no mortality or observable clinical toxicity at doses up to 2000–5000 mg/kg, supporting high tolerability in acute exposure scenarios [48, 50].

Repeated‐dose studies further support the generally favorable safety profile of turmeric‐derived preparations. Subchronic administration of turmeric essential oil, NR‐INF‐02 polysaccharide extract, and CURCUGEN for periods ranging from 28 to 90 days produced no treatment‐related adverse effects in hematological, biochemical, or histopathological parameters, with NOAEL values reaching 1000–2000 mg/kg/day in some cases [39, 49, 51, 52]. Comparable findings were reported for curcumin‐based formulations, including solid lipid particles and curcuminoid–essential oil complexes, which showed no significant toxicity following repeated oral administration in rodents [54, 57]. In addition, bisdemethoxycurcumin and tetrahydrocurcuminoids demonstrated favorable safety profiles supporting the use in food supplements [58, 59].

Despite this overall pattern of safety, some studies indicate that high dietary concentrations or prolonged exposure to turmeric extracts may produce mild hepatic alterations. For example, long‐term administration of turmeric powder at 5% of the diet induced reduced body weight gain, changes in liver weight indices, and histopathological evidence of focal hepatic necrosis and regeneration in rodents [12]. Likewise, elevated liver weights and mild fatty degeneration were observed in rats receiving high doses of curcuminoids over extended periods, although most biochemical parameters remained within normal physiological ranges [47]. However, the mechanistic interpretation of these hepatic findings remains limited, as the available studies do not assess pathways such as oxidative stress, inflammatory signaling, or metabolic enzyme modulation in a systematic manner. As a result, it is not yet possible to establish a unified mechanism linking the observed hepatic responses across different extract types and dosing regimens.

A deeper biochemical interpretation points to the role of curcuminoids in modulating Phase I and II detoxification enzymes, mitochondrial function, and inflammatory mediators. While curcumin is known to activate nuclear factor erythroid 2–related factor 2 (Nrf2) [60] and suppress nuclear factor kappa B (NF‐κB) [61], excessive activation or prolonged exposure may paradoxically impair cellular resilience, especially in metabolically active tissues like the liver.

Additional biochemical alterations, including reductions in serum protein and increases in urea levels, have also been reported following repeated exposure to ethanolic turmeric extracts [53]. Together, these findings suggest that hepatic responses may emerge primarily under high‐dose or prolonged exposure conditions. However, many of these effects occurred at exposure levels that exceed typical human intake, highlighting the importance of dose relevance when interpreting preclinical toxicity signals.

Differences in toxicity outcomes among studies may partly reflect variations in extract composition, formulation strategies, and systemic bioavailability. Preparations enriched with specific fractions, such as polysaccharide‐based extracts or lipid‐enhanced curcumin formulations, frequently demonstrated improved tolerability in repeated‐dose studies [49, 54, 57]. These differences underscore the need for greater mechanistic clarity regarding how formulation‐dependent changes in absorption and metabolism modulate tissue‐level responses, particularly in the liver. Without such integrated data, cross‐study comparisons remain largely descriptive.

The current body of evidence has several strengths, including the use of multiple rodent species, extended exposure periods, and the application of standardized toxicological protocols in several studies, including OECD‐compliant evaluations [56, 58]. Nevertheless, important limitations remain. Dose selection varied widely across studies, with some investigations employing extremely high exposure levels that exceed typical human consumption. In addition, differences in extract standardization, study duration, and reporting of histopathological findings complicate direct cross‐study comparisons. Another limitation is the absence of integrated assessments linking systemic exposure (e.g., plasma levels of curcuminoids) with tissue responses, which limits the interpretation of dose–response relationships and biological probability across models.

From a translational perspective, the available data suggest that turmeric extracts and curcumin‐based formulations are generally well tolerated within experimentally established dosing ranges. However, the occurrence of mild hepatic changes at high dietary concentrations highlights the importance of dose optimization and standardized formulations when translating preclinical findings to human applications. Future studies should prioritize long‐term safety evaluations using physiologically relevant doses, deeper characterization of extract composition, and incorporation of pharmacokinetic measurements to link exposure with observed tissue effects. Such approaches would help develop a clearer mechanistic framework for understanding the spectrum of acute to chronic toxicity across different formulations and biological systems.

## 4. Integrative Perspectives on Toxicological Evaluation and Clinical Translation of Turmeric

### 4.1. Formulation‐Dependent Toxicological Variability of Turmeric‐Derived Products

The toxicological profile of *C. longa* preparations is strongly influenced by extraction methods, chemical composition, and delivery systems. Turmeric products evaluated in preclinical studies include crude or solvent extracts, essential oils, polysaccharide fractions, standardized curcuminoid mixtures, lipid‐based formulations, and nanoparticle delivery systems. These preparations differ considerably in bioavailability, metabolic stability, and tissue distribution, which in turn can influence both therapeutic efficacy and toxicity. For example, polysaccharide‐rich extracts such as NR‐INF‐02 and lipid‐based curcumin formulations have demonstrated favorable safety profiles in repeated‐dose animal studies, showing no treatment‐related alterations in clinical chemistry, hematology, or histopathology even at relatively high doses [48, 49, 54]. Similarly, turmeric essential oil did not produce systemic toxicity or genotoxicity in rodents following repeated oral administration [39].

Conversely, certain high‐concentration extracts or poorly standardized preparations have been associated with biochemical alterations indicative of hepatic stress, including changes in liver weight indices or mild histopathological findings during prolonged exposure [12, 47, 53]. Differences in toxicity among formulations likely reflect variations in curcuminoid concentration, the presence of additional phytochemicals, and enhanced cellular uptake associated with advanced delivery systems such as nanoparticles [28]. Nanoparticle‐based formulations can significantly increase cellular internalization and systemic bioavailability of curcuminoids, potentially elevating intracellular concentrations and altering tissue distribution. These pharmacokinetic changes may influence the safety profile of such formulations and therefore warrant formulation‐specific toxicological evaluation.

### 4.2. Dose–Response Relationships and Safety Thresholds

Across experimental studies, turmeric extracts and their principal constituents demonstrate relatively low acute toxicity, with reported LD_50_ values frequently exceeding 2000–5000 mg/kg body weight in rodents [39, 48, 52, 55]. Subchronic and chronic exposure studies generally report high tolerability, with NOAELs ranging between approximately 720 and 2000 mg/kg/day depending on the tested formulation and study design [49, 52, 54, 58]. These findings indicate a broad therapeutic margin for turmeric‐derived compounds in experimental models.

Nevertheless, some studies suggest that prolonged exposure to very high dietary concentrations may induce adaptive physiological responses, particularly within hepatic tissue. For instance, long‐term dietary administration of turmeric powder has been associated with reduced body weight gain and histopathological evidence of focal hepatic necrosis in rodents [12], while elevated liver weights and mild fatty degeneration have been reported following extended exposure to high doses of curcuminoids [47]. Such effects may reflect metabolic adaptation or enzyme induction rather than overt toxicity, yet they emphasize the importance of establishing clearly defined upper safety thresholds for chronic use.

### 4.3. Species‐Specific Differences and Translational Considerations

Animal models provide essential information regarding systemic toxicity, reproductive effects, and dose–response relationships; however, interspecies differences can complicate the extrapolation of these findings to human populations. Rodents differ from humans in several physiological processes relevant to xenobiotic metabolism, including cytochrome P450 enzyme expression, intestinal absorption mechanisms, and gut microbiota composition. These factors may influence the biotransformation, bioavailability, and clearance of curcuminoids, thereby affecting toxicity outcomes [62–64].

Furthermore, many experimental studies employ exposure levels far exceeding typical human dietary intake of turmeric, potentially exaggerating toxicological responses. As a result, translational interpretation of preclinical data requires careful consideration of pharmacokinetic parameters and physiologically relevant exposure levels. The integration of human‐relevant experimental systems, such as hepatocyte cultures, organoid models, or humanized animal models, may improve predictive accuracy and support safer clinical translation of turmeric‐derived therapeutics.

### 4.4. Regulatory and Safety Perspectives

Several international regulatory agencies have evaluated the safety of curcumin and related compounds. The JECFA established an acceptable daily intake for curcumin of 0–3 mg/kg body weight based on toxicological and reproductive studies in rodents [36]. Similarly, the EFSA assessed tetrahydrocurcuminoids as a novel food ingredient and concluded that intake levels up to 2 mg/kg body weight/day are safe for adults when consumed as dietary supplements [59].

These regulatory limits reflect a conservative risk‐assessment approach that accounts for uncertainties in long‐term exposure and interindividual variability. Although experimental studies often report safety at substantially higher doses, regulatory agencies apply large safety factors when translating animal data to human dietary recommendations. As novel formulations designed to enhance curcumin bioavailability continue to emerge, ongoing reassessment of these safety thresholds may be necessary.

### 4.5. Current Knowledge Gaps and Priorities for Future Research

Despite a growing body of toxicological evidence, several important knowledge gaps remain. Long‐term carcinogenicity studies for many turmeric constituents are limited, and the genotoxic potential of newly developed delivery systems has not been comprehensively evaluated. In addition, interactions between turmeric‐derived compounds and conventional pharmaceuticals remain insufficiently characterized, despite evidence that curcuminoids can modulate drug‐metabolizing enzymes and cellular signaling pathways [65].

Another critical limitation is the insufficiency of safety data in vulnerable populations. Most experimental studies are conducted in healthy adult animals, whereas information regarding the effects of turmeric supplementation during pregnancy, childhood, or in individuals with hepatic or metabolic disorders remains limited. Future research should therefore prioritize chronic exposure studies, standardized extract characterization, pharmacokinetic investigations, and multiorgan toxicity assessments to better define safe exposure ranges.

### 4.6. Clinical Implications and Risk–Benefit Considerations

Turmeric and its curcuminoid constituents have attracted considerable interest due to their anti‐inflammatory, antioxidant [13], antimicrobial [66], and anticancer [67] properties. Experimental evidence indicates that many turmeric‐derived preparations are well tolerated within recommended dosage ranges, supporting their continued exploration as nutraceutical or therapeutic agents [54, 57]. However, the dose‐dependent biological activity of curcuminoids also suggests that excessive or prolonged intake may produce unintended physiological effects, particularly in metabolically active organs such as the liver [12, 47].

For clinical practice, these findings emphasize the importance of considering formulation type, dosage, and individual patient characteristics when recommending turmeric‐based supplements. Patients with underlying hepatic disorders or those receiving medications metabolized by hepatic enzymes may require additional caution. Monitoring biochemical markers such as liver enzyme activity could provide an additional safeguard during long‐term supplementation.

### 4.7. Mechanistic Integration and System Toxicology Perspective

At the molecular level, curcuminoids interact with multiple cellular signaling pathways involved in oxidative stress regulation, inflammation, apoptosis, and DNA damage responses [1, 25, 68]. Curcumin has been shown to activate the Nrf2 pathway, promoting antioxidant defense mechanisms, while simultaneously inhibiting NF‐κB signaling, thereby reducing inflammatory responses [60, 61].

Importantly, these same pathways underlie both the pharmacological and toxicological actions of turmeric and its main components: At dietary or therapeutic exposure levels, the modulation of redox balance, mitochondrial function, and cell‐death signaling contributes to antioxidant, anti‐inflammatory, and cytoprotective effects, whereas at high experimental or supraphysiological doses, the balance may shift toward oxidative injury, mitochondrial dysfunction, apoptosis, and genotoxic stress, particularly in sensitive developmental and reproductive contexts. This dose‐ and context‐dependent behavior helps to explain the apparently dual nature of biological effects of this herb and its main components reported across experimental models [53, 69].

A systems toxicology framework integrating transcriptomic, proteomic, and metabolomic data could provide deeper insight into the network‐level responses triggered by turmeric compounds. Such approaches may facilitate the identification of early biomarkers of toxicity, clarify dose‐dependent molecular responses, and support the development of personalized strategies for the safe therapeutic use of turmeric‐derived products.

### 4.8. Polypharmacological Properties and Their Potential Contribution to Toxicity

*C. longa* constituents are widely recognized for their polypharmacological behavior, meaning that they can simultaneously modulate multiple molecular targets. While this property underlies many of their reported biological effects, it may also contribute to the higher toxicity potency observed in certain settings. By affecting several pathways at the same time, including redox‐regulating systems, inflammatory mediators, apoptosis‐related signaling, and metabolic enzymes, these compounds may generate convergent or overlapping stress responses that exceed the adaptive capacity of the cells. This is consistent with the context‐dependent shift of curcuminoids from antioxidant to pro‐oxidant activity, suggesting that multitarget engagement could amplify susceptibility to toxicity under conditions of high dosing or compromised metabolic balance. Although additional mechanistic studies are needed, acknowledging this system‐level interaction provides a more comprehensive explanation of the observed toxicological outcomes.

### 4.9. Structure–Activity Relationship Considerations in Toxicity of *C. longa* Constituents

Although the toxicological findings across pregnancy‐related, mutagenic, genotoxic, and acute‐to‐chronic toxicity studies point to dose‐dependent and context‐specific biological effects, the structure–activity relationship of the main chemical groups in *C. longa* provides an additional viewpoint for interpreting these outcomes. Curcuminoids such as curcumin, demethoxycurcumin, and bisdemethoxycurcumin share two structural motifs that recur across the studies reporting oxidative stress, apoptosis, or DNA‐damage signaling: the α, β‐unsaturated carbonyl chain and multiple phenolic hydroxyl groups. The α‐, β‐unsaturated carbonyl system can serve as a weak Michael acceptor, a property consistent with observations of ROS elevation, mitochondrial apoptotic signaling, and caspase‐3 activation in blastocysts and oocytes exposed to micromolar curcumin concentrations [29, 30]. Similarly, the redox‐active phenolic groups may contribute to the ROS‐associated responses seen in embryonic models at higher doses [29], as well as to the oxidative stress–linked DNA damage markers, such as phospho‐H2AX and p53 induction in HT1080 cells [43]. However, these interactions have been demonstrated mainly under in vitro conditions using concentrations above those typically achieved in vivo, and several animal studies report an absence of teratogenicity or systemic toxicity even at high oral doses [35, 36, 38], indicating that intrinsic reactivity alone does not predict whole‐organism outcomes.

In contrast, the major constituents of turmeric essential oil, including ar‐turmerone and related sesquiterpenes, lack strongly electrophilic centers and do not contain α, β‐unsaturated carbonyl groups. This structural difference aligns with the repeated absence of mutagenicity or genotoxicity in bacterial and mammalian assays using essential oil [39], and the lack of developmental toxicity in several in vivo models unless extremely high extract doses are administered. Likewise, curcuminoid analogs that have undergone hydrogenation, such as tetrahydrocurcuminoids, exhibit reduced electrophilicity due to the saturation of the conjugated system and show no genotoxic concerns in standardized toxicity tests [59], further supporting the contribution of the unsaturated carbonyl motif to stress‐associated cellular responses.

Formulation‐dependent changes also appear relevant. Nanoparticle‐based curcumin systems do not alter the electrophilic or phenolic functionalities of the molecule, yet they can increase systemic availability, which may shift dose thresholds without changing the structure–activity relationship itself. This is consistent with observations that curcumin nanoparticles behave as indirect mutagens only after metabolic activation [28], and that C‐LNCs do not induce developmental toxicity at doses where free curcumin is known to affect embryonic and oocyte outcomes [38].

Together, these findings suggest that the α, β‐unsaturated carbonyl chain and phenolic hydroxyl groups present in curcuminoids may contribute to some of the oxidative stress, apoptosis, and DNA damage–related effects observed in vitro, whereas constituents lacking these features, such as turmerones, show limited evidence of comparable activity. Nonetheless, the variability of doses, model systems, and formulation strategies across available studies indicates that structure–activity relationship‐based interpretations should remain cautious, and future work comparing defined structural variants across harmonized in vitro and in vivo conditions would strengthen mechanistic conclusions.

### 4.10. Study Bias and Interpretation Limitations

Despite the breadth of available data on *C. longa* toxicity, several sources of bias may influence the interpretation of results. Publication bias represents a potential concern, as studies reporting safety or therapeutic benefits may be more likely to be published than those identifying adverse outcomes. Experimental bias may also arise from methodological limitations, including inconsistent dosing protocols, inadequate randomization or blinding, and selective reporting of endpoints. Species‐related bias is inherent in preclinical toxicology studies, as rodent models may not fully replicate human metabolic, pharmacokinetic, or immunological responses. In addition, formulation‐related bias may occur when specific delivery systems receive disproportionate research attention due to commercial development interests, potentially shaping the perceived safety profile toward well‐funded formulations. Recognizing these limitations is essential for interpreting toxicological findings and for guiding future investigations toward more balanced, reproducible, and clinically relevant evidence.

## 5. Conclusion

The toxicological evaluation of *C. longa* and its major constituents and formulation‐derived variants, including curcuminoids (such as curcumin, demethoxycurcumin, and bisdemethoxycurcumin), hydrogenated curcumin analogs (e.g., tetrahydrocurcuminoids), turmeric essential oil components (such as ar‐turmerone), and nano‐ or lipid‐based curcumin formulations, reveals a detailed safety profile shaped by dose, formulation, and metabolic context. While most turmeric‐based compounds demonstrate low acute toxicity and favorable genotoxic and mutagenic outcomes under standard conditions, specific formulations and high‐dose exposures can elicit hepatocellular stress, DNA damage, or metabolic adaptation. Mechanistic insights point to oxidative stress, p53 activation, and mitochondrial modulation as central pathways underlying these effects. Importantly, formulation strategy, whether lipid‐based, polysaccharide‐rich, or nanoparticle‐enhanced, plays a decisive role in modulating both therapeutic efficacy and toxicological risk.

Methodological inconsistencies, species‐specific sensitivities, and predictive limitations across studies underscore the need for harmonized protocols and human‐relevant models. Recognizing potential biases and refining experimental controls will be critical for advancing reproducible and clinically meaningful safety assessments. From a translational standpoint, turmeric‐derived therapeutics appear safe within defined exposure thresholds, but their integration into clinical practice demands precision dosing, formulation‐specific evaluation, and ongoing monitoring, particularly in vulnerable populations or chronic‐use scenarios.

Future research should prioritize systems‐level toxicology, comparative formulation studies, and biomarker development to guide personalized risk assessment. By bridging mechanistic depth with regulatory and clinical insight, the therapeutic promise of *C. longa* can be safely and effectively realized.

## Author Contributions

JJ raised the notion and helped in gathering the data. MGR wrote the manuscript.

## Funding

The authors received no financial support for the research, authorship, and/or publication of this article.

## Disclosure

All authors approved the final version of the manuscript.

## Ethics Statement

The authors have nothing to report.

## Consent

The authors have nothing to report.

## Conflicts of Interest

The authors declare no conflicts of interest.

## Data Availability

Data sharing is not applicable to this article as no datasets were generated or analyzed during the current study.
